# Case report: CT features of cherubism

**DOI:** 10.4103/0971-3026.38506

**Published:** 2008-02

**Authors:** Avinash R Kambadakone, Rajgopal V Kadavigere, Raghu R Hosahalli, Sudha S Bhat

**Affiliations:** Department of Radiodiagnosis and Imaging, Kasturba Medical College, Manipal, India; 1 Department of Pathology, Kasturba Medical College, Manipal, India

**Keywords:** Cherubism, CT, fibrossseous neoplasms, mandible, maxilla

Cherubism is a rare hereditary disease of non-neoplastic origin seen in childhood and characterized by bilateral bony enlargement of the jaws.[1] It causes diffuse, bilateral, and multilocular expansion of the mandible and/or maxilla and has a characteristic radiographic and histopathological appearance.[1,2] In this case report, we describe the CT features of this uncommon condition in a 14-year-old girl.

## Case Report

A 14-year-old girl presented with a history of painless swelling of the jaws since 5 years of age. The swelling was initially in the region of the parotid and then slowly progressed to involve both sides of the mandible. The patient did not reveal any history of pain, visual disturbances, or dental complaints. Clinical examination revealed enlargement of the jaws and cheeks by a hard mass, which was seen to involve the body of the mandible and the rami. There was no local tenderness. Mouth opening was adequate, with a ‘V’-arched palate on intraoral examination. Submandibular lymph nodes were palpable bilaterally. Past medical and family histories were unremarkable. Hematological and biochemical evaluation, including serum calcium and serum phosphorus concentrations and alkaline phosphatase activity, were within normal limits.

**Figure 1 (A, B):**
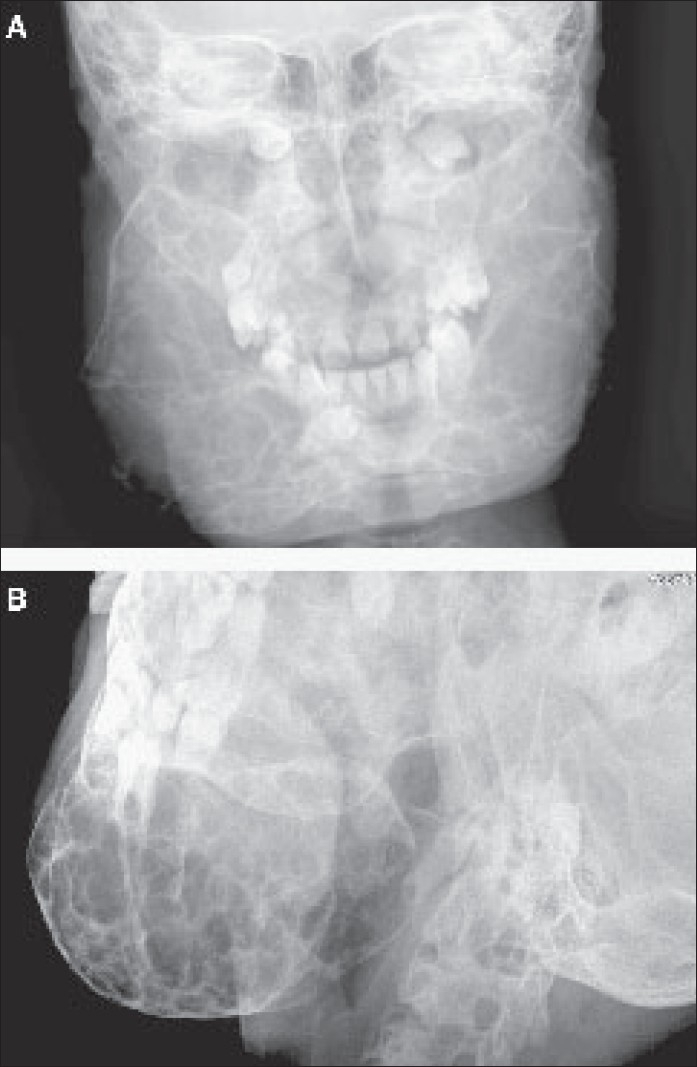
Frontal (A) and oblique (B) radiographs show expansile remodeling of the maxilla and mandible with multilocular radiolucent lesions causing cortical thinning

**Figure 2:**
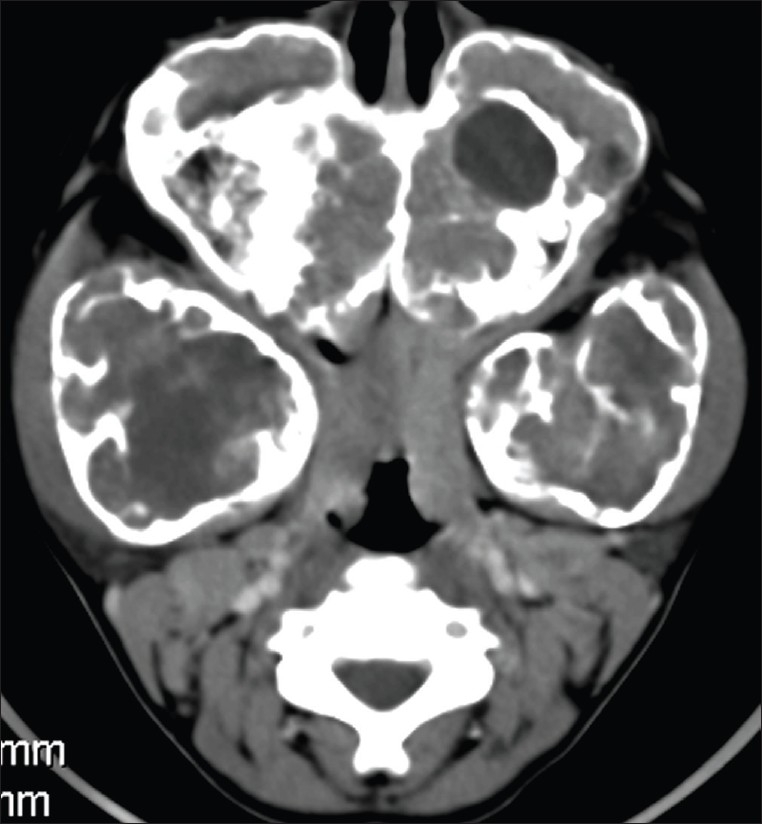
Axial CT image shows expansile lesions in the mandible and the maxilla, with mildly sclerotic matrix and internal trabeculations

**Figure 3:**
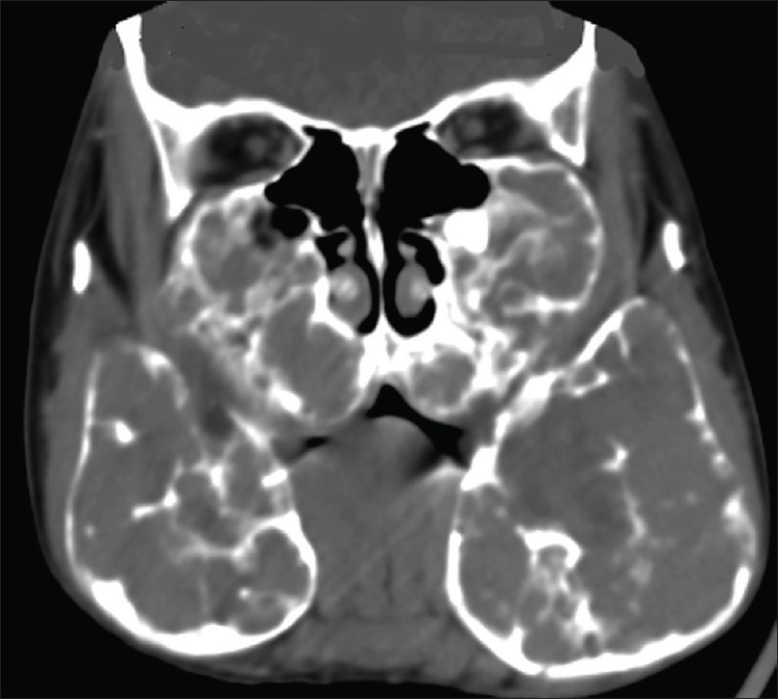
Coronal CT scan reveals expansile maxillary lesions with obliteration of the antral cavity and extension to the floor of the orbit

**Figure 4:**
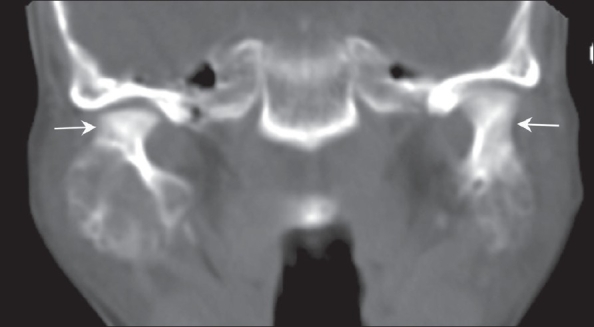
Coronal CT scan reveals sparing of both mandibular condyles (arrows)

**Figure 5:**
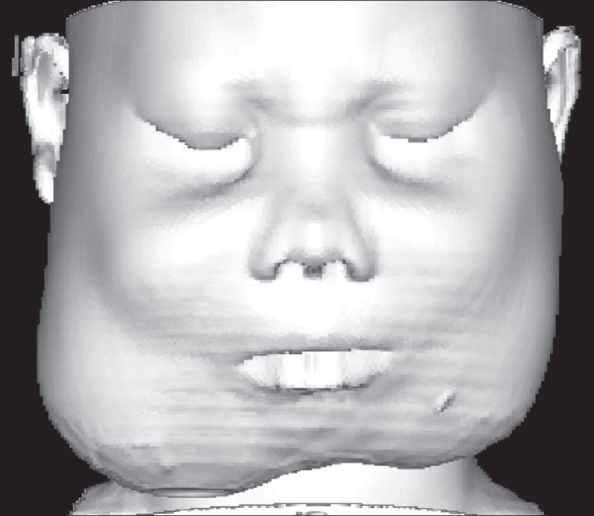
Three-dimensional surface rendering shows expansile osseous remodeling of the jaws

Radiographic examination revealed bilateral, multilocular, and radiolucent lesions with a ground-glass appearance extensively involving the maxilla and mandible. The mandibular lesions showed expansile osseous remodeling with thinned cortical rims. There was involvement of the body and ramus [Figure 1]. There was consequent obliteration of the maxillary antrum, with displacement of the unerupted teeth up to the floor of the orbit. A CT scan showed extensive expansile remodeling of the maxilla and the mandible, with internal trabeculations and a mildly sclerotic matrix [Figure 2], causing obliteration of the maxillary antrum, with encroachment onto the orbital floor [Figure 3], and consequent bilateral proptosis, which was seen clinically. There was no cortical break, fracture, or periosteal reaction seen within the jaw bones. Bilateral condylar extension was characteristically absent [Figure 4]. CT also clearly depicted the absence of extraosseous soft tissue extension. Three-dimensional shaded-surface rendering showed the characteristic cherubic appearance [Figure 5].

**Figure 6:**
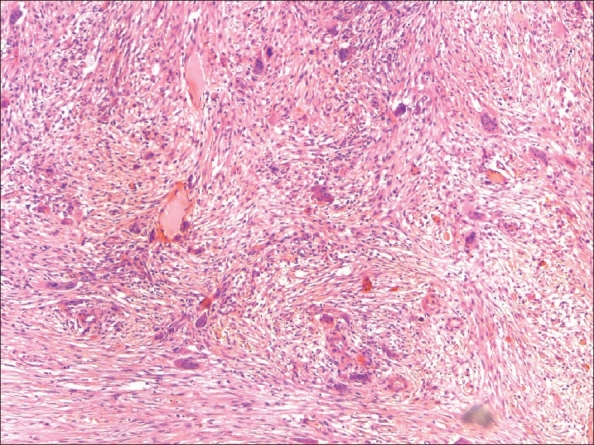
Photomicrograph shows numerous multinucleated giant cells and vascular spaces scattered within a fibrous connective tissue stroma (hematoxylin and eosin stain; original magnification ×100)

The patient underwent cosmetic shavings of the mandibular lesions. Intraoperatively, multiple cystic cavities filled with cheesy material were found within the mandibular lesions. On gross examination of the cut sections, gray-white areas, cartilaginous areas, and hemorrhagic areas were seen. On microscopy, long spicules of lamellar bone lined by osteoblasts were seen. The marrow spaces were filled with dense spindle cell proliferation, with fascicles of spindle cells arranged in a storiform pattern. Numerous multinucleate osteoclastic giant cells were dispersed throughout the lesion, with focal clustering around areas of hemorrhage [Figure 6]. Areas of dense fibrosis were also seen.

## Discussion

Cherubism, an inherited, fibroosseous condition, is characterized by firm, painless swellings of the jaw, producing a cherub-like appearance. It was first described in 1933 by Jones as ‘familial multilocular cystic disease of jaws,’ but the term ‘cherubism’ was later coined by him in 1948 to describe the classical characteristics of full round cheeks, which resembled those of the cherubs immortalized by Renaissance art.[3–5] The condition was initially described as hereditary, but both hereditary and sporadic cases have been described.[5] Cherubism is transmitted as an autosomal dominant trait, with greater penetrance in males (100%) than in females (50-70%).[6] The enlargement of the jaw bones occurs as an isolated finding and affected children do not have other mental or physical abnormalities.[2] However, it has been occasionally associated with other genetic disorders, such as Noonan-like syndrome or Noonan-like/ multiple giant-cell-like lesion syndrome.[2]

It presents in the first few years of life with bilateral expansion of the mandible and/or maxilla, becoming increasingly evident at the time of puberty.[2] It shows gradual regression thereafter and usually involutes by middle age.[1,2] The symptoms and signs depend on the severity of the condition and range from mild to severe deformity of the jaws - sometimes even causing respiratory distress due to complete nasal obstruction.[7,8] Dental abnormalities are seen, with serious disturbances in dentition, including premature loss of deciduous teeth and displaced, unerupted, or absent permanent teeth with features of malocclusive dentition.[2,7,9] The patient in our report presented with painless swelling of the jaws.

The diffuse, bilateral, and multilocular nature of this condition is distinctive radiographically, beginning at the angle of mandible and extending into the ramus and body.[1,2] Radiologically, the unique features of cherubism include well-defined, mild to extensive, multilocular areas of reduced density, with a few intervening irregular bony septae and expansile remodeling and thinning of the cortical rims.[2,5] About 60-70% of the lesions may be mildly sclerotic and upto 70% may demonstrate internal trabeculae.[5] These multilocular areas of diminished densities are later replaced by irregular patchy sclerosis, with progressive calcification.[2] The classical ground-glass appearance of the lesions is a result of the presence of small tightly compressed trabeculae.[2] Seward and Hankey[10] have proposed a grading system based on the radiographic location of the lesions in the jaws. It is as follows:

Grade I: Involvement of bilateral mandibular molar regions and ascending rami, mandible body, or mentis.

Grade II: Involvement of bilateral maxillary tuberosities (in addition to grade 1 lesions) and diffuse mandibular involvement.

Grade III: Massive involvement of the entire maxilla and mandible, except the condyles.

Grade IV: Involvement of both jaws, including the condyles.

According to this grading system, our patient showed Grade III disease.

CT clearly depicts the extent of the mandibular and maxillary lesions as compared to plain radiographs; the anatomic complexity makes interpretation of radiographs of the facial bones difficult.[5] A fibroosseous, mildly sclerotic matrix is typically seen, with expansile remodeling of the bone and cortical thinning.[5] The lesions in our patient showed a mildly sclerotic matrix. Some reports have also documented soft-tissue density material on CT.[11] In our patient, none of the osseous lesions showed adjacent periosteal reaction or an associated soft-tissue mass. Though sparing of the mandibular condyles has been described as a pathognomonic feature of cherubism, condylar involvement is often seen on CT scan.[12] Condylar involvement was, however, absent in our patient. Encroachment onto the orbital floor from the maxillary lesions was also well seen on the coronal CT reconstructions, but there was no involvement of the orbital structures.

There are very few reports describing the MR features of cherubism,[5,11] which are usually nonspecific. The lesions have been described as being homogenously isointense or heterogeneous on T1W images and heterogeneously isointense or hyperintense on fat-suppressed spin-echo T2W images.[5,11]

Microscopically, the lesions show numerous multinucleated giant cells and vascular spaces which are randomly distributed against a background of fibrous connective tissue.[2] Histochemical and immunohistochemical characterization of the multinucleated giant cells reveals that these are osteoclasts since they are positive for tartrate-resistant acid phosphatase and express the vitronectin receptor.[2] The stroma is moderately collagenous and contains focal deposits of hemosiderin pigment. Eosinophilic collagen perivascular cuffing can be seen in some cases and this perivascular hyalinolysis is considered pathognomonic for cherubism.[13,14]

Conditions like craniofacial fibrous dysplasia, brown tumor of hyperparathyroidism, and familial gigantiform cementoma can present with radiographic features similar to that of cherubism and should be considered in its differential diagnosis.[5] Cherubism and craniofacial fibrous dysplasia can be distinguished clinically and radiographically. Cherubism is characterized by bilateral mandibular involvement, limitation to the maxilla and mandible, and involution at the time of puberty.[5] The clinical features of cherubism, like swollen cheeks, upward turning of the eyes, and dental derangement, are typically not present in fibrous dysplasia. Histologically, numerous multinucleated giant cells are seen in patients with cherubism, while they are rarely seen in fibrous dysplasia. Brown tumor and Jaffe-Campanacci syndrome can be readily distinguished on clinical grounds. Familial gigantiform cementoma is a rare osseous disorder in which the lesions are primarily located in the maxilla and cause focal enlargement rather than diffuse involvement.[5] Histologically, multinucleated giant cells are absent and the lesions contain cementum.[5]

Surgical intervention may be undertaken in patients with serious cosmetic or functional problems. Since the lesions undergo spontaneous regression it is better if surgical intervention is delayed until after puberty. Curettage or surgical contouring, with or without bone grafting, is the treatment of choice.[2]

Though the clinical and radiographic characteristics of cherubism strongly suggest the diagnosis, a CT study helps in the accurate evaluation of the osseous extension of the lesions. CT is particularly beneficial for assessing the extension of the lesions into the orbit.
